# Intensity-Modulated Interventional Radiotherapy (Modern Brachytherapy) Using 3D-Printed Applicators with Multilayer Geometry and High-Density Shielding Materials for the NMSC Treatment

**DOI:** 10.3390/jpm15100460

**Published:** 2025-09-30

**Authors:** Enrico Rosa, Sofia Raponi, Bruno Fionda, Maria Vaccaro, Antonio Napolitano, Valentina Lancellotta, Francesco Pastore, Gabriele Ciasca, Frank-André Siebert, Luca Tagliaferri, Marco De Spirito, Elisa Placidi

**Affiliations:** 1 UOC Fisica per le Scienze della Vita, Dipartimento di Diagnostica per Immagini, Radioterapia Oncologica, Fondazione Policlinico Universitario Agostino Gemelli IRCCS, 00168 Rome, Italy; 2 Department of Theoretical and Applied Sciences, Università Telematica eCampus, 22060 Novedrate, Italy; 3 Sezione di Fisica, Università Cattolica del Sacro Cuore, 00168 Rome, Italy; 4 UOC Degenze di Radioterapia Oncologica, Dipartimento di Diagnostica per Immagini e Radioterapia Oncologica, Fondazione Policlinico Universitario A. Gemelli IRCCS, 00168 Rome, Italyvalentina.lancellotta@policlinicogemelli.it (V.L.);; 5 Medical Physics Unit, Bambino Gesù Children’s Hospital, 00165 Rome, Italy; 6 Clinic of Radiotherapy, University Hospital of Schleswig-Holstein, Campus Kiel, 24105 Kiel, Germany; 7 Dipartimento di Scienze Radiologiche ed Ematologiche, Università Cattolica del Sacro Cuore, 00168 Rome, Italy

**Keywords:** interventional radiotherapy, NMSC, 3D-printing, personalized radiotherapy

## Abstract

**Background/Objectives**: This study investigates the dosimetric impact of a 3D-printed applicator integrating multilayer catheter geometry and high-density shielding, designed for contact interventional radiotherapy (IRT) in non-melanoma skin cancer (NMSC) treatment. The aim is to assess its potential to enhance target coverage and reduce doses in organs at risk (OARs). **Methods**: A virtual prototype of a multilayer applicator was designed using 3D modeling software and realized through fused deposition modeling. Dosimetric simulations were performed using both TG-43 and TG-186 formalisms on CT scans of a water-equivalent phantom. A five-catheter array was reconstructed, and lead-cadmium-based alloy shielding of varying thicknesses (3–15 mm) was contoured. CTVs of 5 mm and 8 mm thickness were analyzed along with a neighboring OAR. Dosimetric endpoints included V95%, V100%, V150% (CTV), D_2cc_ (OAR), and therapeutic window (TW). **Results**: Compared to TG-43, the TG-186 algorithm yielded lower OAR doses while maintaining comparable CTV coverage. Progressive increase in shielding thickness led to improved V95% and V100% values and a notable reduction in OAR dose, with an optimal trade-off observed between 6 and 9 mm of shielding. The TW remained above 7 mm across all configurations, supporting its use in lesions thicker than conventional guidelines recommend. **Conclusions**: The integration of multilayer catheter geometry with high-density shielding in a customizable 3D-printed applicator enables enhanced dose modulation and OAR sparing in superficial IRT. This approach represents a step toward personalized brachytherapy, aligning with the broader movement in radiation oncology toward patient-specific solutions, adaptive planning, and precision medicine. Future directions should include prototyping and mechanical testing of the applicator, experimental dosimetric validation in phantoms, and pilot clinical feasibility studies to translate these promising in silico results into clinical practice.

## 1. Introduction

Non-melanoma skin cancer (NMSC) is the most frequent malignancy worldwide, and its incidence continues to rise, particularly in elderly populations. Interventional radiotherapy (IRT, or modern brachytherapy, BT) represents a highly effective treatment modality for NMSC, offering excellent cosmetic outcomes, precise dose delivery, and the possibility of hypofractionated outpatient regimens, making it especially suitable for frail or surgically unfit patients [1,2]. Compared to external beam radiotherapy, IRT provides a more localized treatment with a rapid dose fall-off, thereby sparing adjacent healthy tissues and critical structures. This dosimetric characteristic is particularly advantageous in the management of lesions located in anatomically complex regions, where organ preservation and cosmetic considerations are of primary importance. In particular, contact IRT has demonstrated a strong clinical role in the treatment of superficial lesions located in functionally or cosmetically sensitive areas such as the face, where surgical approaches may be disfiguring. Furthermore, clinical evidence highlights high local control rates and favorable toxicity profiles, with minimal acute and late side effects, which contribute to maintaining patients’ quality of life [3,4].

Recent advances in treatment planning and imaging have enabled a more accurate definition of target depth and volume, expanding the therapeutic indications of contact IRT to include lesions potentially exceeding 5 mm in thickness in cases of experienced centers and anatomical locations with no major concerns for organs at risk (OARs). The integration of three-dimensional imaging modalities, such as CT- and MRI-based planning, has improved the precision of contouring and the assessment of tumor extension, thereby reducing uncertainties in dose delivery. This imaging-guided approach has also allowed for more reliable reconstruction of applicators and catheters, improving the reproducibility of treatment plans and aligning dosimetric evaluation with real patient anatomy. A promising strategy to optimize dose distribution for such challenging cases involves multilayer catheter arrangements, allowing dynamic intensity modulation through different source-to-skin distances. This technical solution not only enhances coverage of deeper or irregularly shaped lesions but also enables individualized optimization according to patient-specific anatomy. Such adaptability is especially relevant for lesions located in anatomically irregular or cosmetically sensitive regions, where uniform coverage is often difficult to achieve with conventional single-layer techniques. This approach significantly widens the therapeutic window (TW), defined as the physical distance between the 100% prescription isodose and the 150% isodose, which represents the threshold beyond which skin toxicity is likely to occur [5].

Concurrently, three-dimensional (3D) printing technologies are emerging as powerful tools for the customization of IRT applicators and templates. Personalized applicators allow for optimal conformance to complex anatomical surfaces, improved catheter positioning, and the potential for better dose conformity [6]. This level of personalization is particularly valuable in superficial or irregular lesions, where standard applicators may not guarantee homogeneous dose distribution or sufficient stability during treatment. In addition, 3D printing facilitates the incorporation of shielding components or multilayer structures directly into the applicator design, enabling more advanced modulation strategies within a single patient-specific device. This technology enables the design and fabrication of patient-specific devices within a short timeframe, using biocompatible and cost-effective materials (e.g., thermoplastics) that can be sterilized and safely introduced into clinical practice. The relatively low production cost and rapid prototyping process make this approach suitable not only for highly specialized centers but also for broader implementation in routine clinical settings. Furthermore, the reproducibility of the process supports standardized workflows across different institutions, which could facilitate broader clinical adoption. Several groups have reported the successful integration of 3D printing in clinical workflows for facial skin cancers, using individualized molds to manage curved or irregular surfaces. In addition, 3D printing opens new opportunities for adaptive brachytherapy, where applicators can be rapidly redesigned to accommodate anatomical changes during treatment, and for educational purposes, by providing realistic phantoms for training and quality assurance [6,7].

The combination of multilayer geometry with shielding materials, such as dense alloys of cadmium and lead integrated into the applicator design, opens new possibilities for both dose modulation and healthy tissue sparing. Shielding inserts can protect critical organs at risk (OARs) while maintaining adequate coverage of the target volume. Such innovations, particularly when guided by pre-treatment imaging, may support the transition from conventional to patient-specific approach, intensity-modulated contact IRT, improving the precision and effectiveness of treatment plans in daily clinical practice. The progressive integration of imaging, applicator design, and advanced planning algorithms could ultimately converge into a fully personalized workflow, where treatments are tailored with high reproducibility and accuracy. This evolution represents a step toward personalized brachytherapy, where applicator design, imaging, and planning are seamlessly integrated to maximize tumor control and minimize toxicity [8,9].

The aim of this study is to explore the dosimetric implications of a theoretical 3D-printed applicator that combines, within a single structure, both a multilayer catheter configuration and variable-thickness shielding elements. This concept was developed to address some of the current challenges in superficial interventional radiotherapy, where achieving adequate target coverage must be balanced against the need to protect adjacent critical structures. Specifically, we investigate the impact of this design on target coverage and on the dose received by OARs, providing insight into its potential clinical advantages and limitations. By simulating different shielding configurations and catheter arrangements, the study seeks to identify conditions that optimize the therapeutic window of multilayer applicator while maintaining flexibility for patient-specific adaptation. In this way, the proposed design may represent a preliminary step toward more personalized, image-guided workflows, although further validation and practical testing will be required before clinical translation.

## 2. Materials and Methods

To assess the device’s practical feasibility, a virtual dedicated prototype was developed to validate the proposed design. The applicator was designed using Rhinoceros 8 (Rhino) 3D modeling software and manufactured using fused deposition modeling technology with an Ultimaker S3 printer (Ultimaker, Utrecht, Netherlands). The printing material was white clear polylactic acid (PLA) (density d = 1.24 g/cm^3^), an approximately water equivalent, cost-effective and easy to handle material, with a 0.4 mm extrusion nozzle, 0.2 mm layer height, and 100% infill density to ensure maximum solidity and consistency. Once the virtual applicator was developed, a dosimetric study was conducted to evaluate its performance within a simulated clinical setup.

A computed tomography (CT) scan of a water-equivalent plastic water slab was acquired using a slice thickness of 0.625 mm to simulate a setup for contact intensity-modulated IRT. Catheter reconstruction and treatment planning were performed in the Oncentra Brachy treatment planning system (Elekta^®^, Stockholm, v. 6.2.3). In silico, a five-catheter multilayer array was reconstructed with 1 cm spacing between parallel catheters within each layer. Three catheters were positioned in the upper layer and two in the lower layer, replicating a configuration previously described in the literature [5]. The multilayer applicator was specifically designed with dedicated grooves and guiding channels to ensure consistent catheter positioning within the stepped geometry thus minimizing the impact of inter-operator variability. This structural feature was conceived to improve reproducibility across different treatment sessions and users, reducing uncertainties that could otherwise affect source placement and dose distribution. The clinical target volume (CTV) was delineated in the CT data sets with two different thicknesses (5 mm and 8 mm). An OAR was contoured 1 mm away from the outer margin of the thicker CTV delineation.

A protective square shielding with varying thicknesses (3, 6, 9, 12, and 15 mm) was contoured to shield the OAR at the distance of 0.5 mm from CTV, one shielding thickness at a time. Additionally, a parallelepiped-shaped applicator was delineated to represent the 3D-printed device, while the slab itself was contoured as the external body.

The source used in this study was the HDR 192Ir source (Elekta ^®^, Stockholm, Sweden) (370 MBq). Each catheter included 25 active dwell positions, spaced 2 mm apart along its length. Dose calculations were performed using both the TG-43 formalism [10] and a collapsed cone convolution algorithm (CCC, standard accuracy level–calculation time: 3 min and 22 s), implemented according to the TG-186 guidelines [11]. Therefore, the associated calculation uncertainty is comparable to that of standard clinical scenarios, which has been reported in the literature to be within approximately 3–5%; this range is generally considered acceptable in routine brachytherapy practice, as it reflects the combined effect of algorithmic assumptions, source calibration, and geometric reproducibility [12,13]. This dual-method approach enabled assessment of dose distribution variations arising from differences in material density and composition within and around the catheter arrangement. To ensure a consistent comparison across configurations and limit excessive skin dose, treatment plans were normalized though modifying F factor so that the 150% isodose line remained tangential to the skin surface, resulting in a fixed V150% (CTV) of 1.5%. This approach was chosen to establish a reproducible baseline condition, allowing the evaluation of dose distribution while minimizing confounding effects related to uncontrolled hotspot escalation. The comparison at fixed V150% between TG-43 and TG-186 was performed only for the unshielded configuration to allow a consistent evaluation of the effect of the calculation algorithms. In the subsequent analysis including shielding, no further normalization was applied, and the natural variation in V150% and other dosimetric parameters was reported.

**Figure 1:**
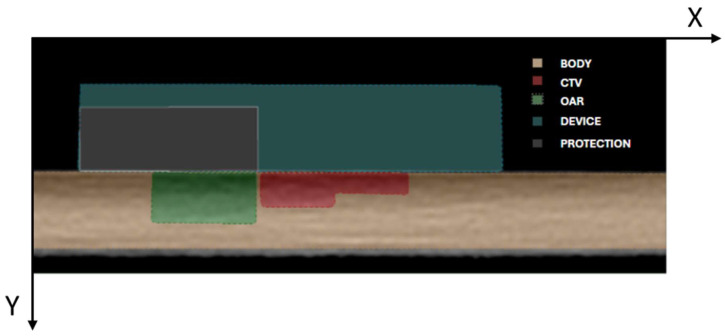
Visualization of the simulation setup including external body (orange), CTV (red), OAR (green), the simulated plastic device (cyan), and attenuating thicknesses (grey).

The attenuating material selected for the simulated study was a lead-cadmium alloy, as AISI316L with a density of 8 g/cm^3^, whereas the simulated device was modeled as uniform Hytrel7246 with a density of 1.26 g/cm^3^. The body and CTV were assigned male soft tissue properties based on Hounsfield Unit (HU) values, while the OAR was characterized as soft tissue according to its HU. All contour delineations are illustrated in Figure 1.

**Figure 2:**
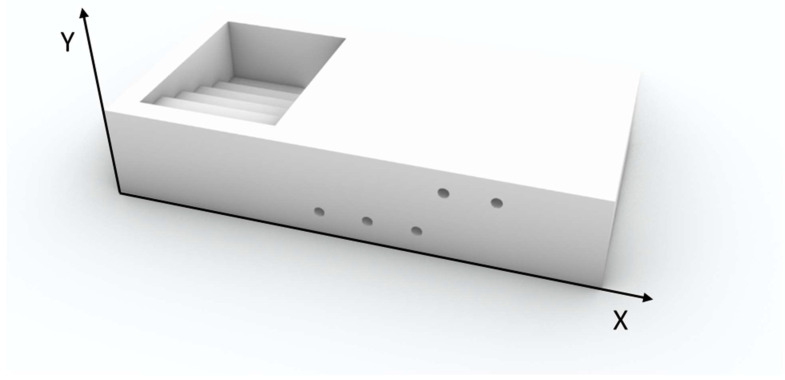
3D renderings of the customized applicator developed from dosimetric evaluations.

**Figure 3:**
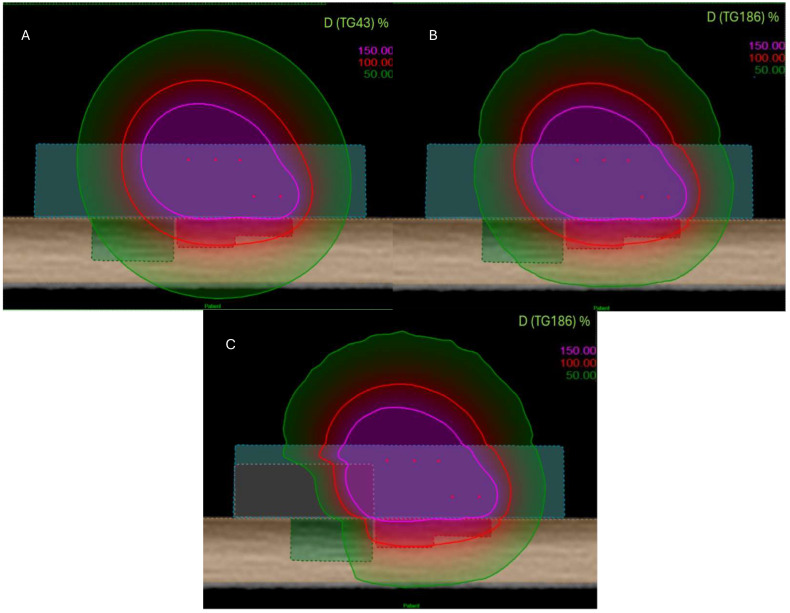
View of dose distribution for three configurations and several algorithms: TG-43 (**A**), TG-186 without shielding (**B**), and TG-186 with 15 mm alloy shielding (**C**). The 150% isodose is shown in purple, the 100% isodose in red, and the 50% isodose in green.

Using the simulation setup described in Figure 1 and the customized applicator illustrated in Figure 2, a dose distributions shown in Figure 3 were obtained. To improve visualization and reproducibility, the central transverse plane passing through the middle dwell positions was selected for the displayed isodose distributions.

To investigate algorithm-dependent differences in dose distribution, the TW was defined, as previously discussed, as the distance between the 100% and 150% isodose lines at the center of the catheter configuration [5]. Finally, dosimetric parameters for the OAR (D2cc, expressed as percentages relative to the prescription dose) and CTV coverage metrics (V95%, V100% and V150%) were analyzed as a function of the shielding thickness.

## 3. Results

As a preliminary step, a dedicated applicator was designed using 3D printing (Figure 2), prior to conducting the dosimetric evaluation. The structure was specifically engineered to accommodate shielding inserts of variable thickness and to ensure practical feasibility for clinical application. The device design (Figure 3) consists of a 3D-printed rectangular structure with external dimensions of 100 mm × 50 mm × 20 mm. The upper section features an open compartment containing a stepped internal scale composed of five levels, each 3 mm in height, for a total elevation of 15 mm. This stepped configuration is intended to house shielding inserts of varying thicknesses.

The lateral walls are perforated with cylindrical holes, 2 mm in diameter, arranged in multiple horizontal rows with both vertical and horizontal spacing set at 10 mm. These channels are specifically designed to hold flex tube catheters, which can be inserted horizontally and anchored at the distal end via a bottom locking element, thus eliminating the need for traditional fixation flaps. Five dedicated grooves are integrated into the structure to guide catheter placement, ensuring accurate and reproducible positioning within the multilayer geometry.

To assess the influence of dose calculation algorithms and shielding configuration, isodose distributions were analyzed in the central plane shown in Figure 1. Figure 3 shows three configurations: TG-43 formalism, TG-186 algorithm without shielding, and TG-186 with a 15 mm thick alloy shielding. The 150%, 100%, and 50% isodose lines (represented in purple, red, and green, respectively) illustrate progressive dose attenuation and redistribution across the configurations.

Dosimetric parameters calculated using the TG-43 and TG-186 formalisms are reported in Table 1. Using the TG-43 formalism, V95% and V100% CTV coverages were 95.73% and 88.19%, respectively, with a V150% of 1.51%. The D2cc dose to the OAR was 79.43%, and the TW was 7.2 mm. Under the TG-186 formalism, the same applicator configuration yielded V95% and V100% values of 92.64% and 84.19%, with an identical V150% of 1.51%. The D2cc OAR dose was reduced to 76.46%, and the TW slightly narrowed to 7.0 mm.

**Figure 4:**
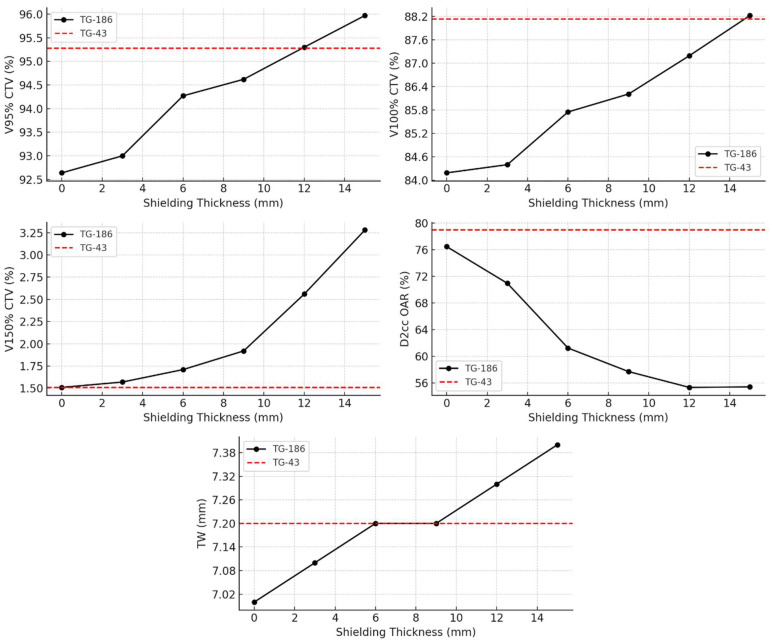
Dosimetric parameters as a function of shielding thickness (0–15 mm) calculated using TG-186. Red dashed lines represent TG-43 values for comparison. CTV coverage (V95%, V100%, V150%), OAR dose (D2cc), and TW are shown separately.

Further analysis under the TG-186 model, presented in Figure 4, evaluated the dosimetric impact of increasing shielding thickness from 0 mm to 15 mm. With thicker shielding, V95% and V100% CTV coverage increased progressively from 92.64% and 84.19% (no shielding) to 95.97% and 88.22% (15 mm shielding), respectively. Simultaneously, the D2cc OAR dose decreased markedly from 76.46% to 55.39%, indicating enhanced sparing of healthy tissue. The V150% rose moderately, from 1.51% to 3.28%, while the TW expanded from 7.0 mm to 7.4 mm. These trends highlight the dose-modulating effect of shielding and are visually represented in Figure 4, where TG-43-based values are shown as dashed red reference lines.

## 4. Discussion

The integration of advanced dose calculation algorithms and the use of patient-specific shielding provide meaningful insight into strategies for dose modulation in IRT. As shown in the comparative analysis, the TG-186 formalism, accounting for tissue heterogeneities, results in a clear reduction of the dose to OAR compared to TG-43, even without the inclusion of shielding. Notably, this reduction in D2cc does not compromise the TW, which remains above 7 mm, thus allowing to theoretically exceed the 5 mm target thickness usually recommended in guidelines for superficial brachytherapy [12].

Interestingly, while V95% and V100% progressively increase with shielding thickness, indicating improved dose coverage of the target volume, V150% follows a different trend. This differing behavior may reflect the fact that, while shielding contributes to a broader and more modulated dose distribution, it can also cause localized dose intensification due to backscatter and attenuation effects, leading to the formation of hotspots. These hotspots may explain the relatively sharper rise in V150% compared to the more uniform increase of V95% and V100%. Therefore, while shielding provides a global improvement in target coverage and OAR sparing, careful evaluation of localized high-dose regions will be required before routine clinical translation, possibly through adaptive planning strategies or real-time dosimetric verification.

Although TG-186 without shielding shows slightly lower CTV coverage compared to TG-43, this limitation can be compensated through individualized plan adaptation, including lesion-specific manual optimization or the use of a thicker bolus. These strategies help restore optimal target coverage while maintaining dose constraints for adjacent organs at risk. Importantly, the progressive introduction of shielding elements leads to a simultaneous enhancement of CTV coverage (V95% and V100%) and a decrease in OAR dose (D2cc), as evidenced by the dose-volume metrics and corresponding graphical trends. This dual benefit demonstrates that shielding improves both target coverage and normal tissue sparing, a particularly valuable outcome in anatomically constrained regions such as the ocular and periocular [14].

These regions represent some of the most challenging scenarios in clinical practice, where organ preservation and cosmetic outcome are equally important as tumor control.

Interestingly, at shielding thicknesses between 6 mm and 9 mm, both CTV coverage and TW values obtained with TG-186 match those of the TG-43 reference. This suggests that the presence of shielding may lead to a compensation between attenuation and backscattering effects, resulting in a reduction between the dose distributions calculated with the two algorithms in terms of target coverage and depth of effective treatment, while still accounting for tissue heterogeneity. A shielding thickness of approximately 12 mm provides optimal V95% coverage to the CTV. Beyond this point, the dose to the OARs begins to rise again, suggesting that further increases in thickness may compromise OAR sparing. This observation confirms the existence of an optimal therapeutic compromise for shielding design, where improvements in coverage and protection coexist, but which can be lost if shielding is excessively increased. This reflects a trade-off between achieving optimal target coverage and maintaining effective protection of surrounding healthy tissues within a compact treatment geometry, unlike earlier assumptions suggesting possible toxicity increase at high shielding thicknesses. This result aligns with previous experiences in superficial and interstitial IRT for head and neck and pelvic regions, including nasal vestibule and rectal cancer, where metallic shielding effectively reduced doses to nearby critical structures such as eye lenses, without compromising CTV coverage [15,16,17,18].

Our findings were compared with previously reported strategies using customized applicators and shielding in contact IRT. Several studies have demonstrated the feasibility of 3D-printed molds to improve dose conformity over curved anatomical surfaces, especially in challenging facial region [19,20]. Regarding shielding, previous work has reported the use of high-density metallic layers, including lead and tungsten, to reduce OAR exposure, typically employing thicknesses in the range of 3–10 mm [14,18]. However, these approaches often relied on rigid or single-thickness inserts, limiting their adaptability to complex or irregular surfaces. Compared to these approaches, our multilayer applicator design enables a more flexible modulation of the shielding effect by integrating stepped thickness levels within a single structure, allowing individualized dose optimization without compromising CTV coverage. This integration of geometry and shielding represents an advancement toward personalized, image-guided contact IRT workflows. Another relevant finding of this work concerns the TW. The ability to combine imaging data, dose calculation algorithms, and customizable applicator design is expected to streamline the treatment planning process and improve reproducibility across different clinical centers. In our simulations, the TW remained above 7 mm across all shielding configurations, which is particularly significant from a clinical perspective. Current clinical guidelines generally recommend a maximum target thickness of approximately 5 mm for safe treatment with contact IRT [15]. Our results suggest that the proposed multilayer applicator, when combined with high-density shielding, may safely extend treatment indications to lesions exceeding this limit, while maintaining adequate CTV coverage and sparing of nearby organs at risk. This potential expansion of indications could have a direct impact on patient management, making contact IRT a feasible option for a broader group of patients, including those with thicker or more irregular lesions traditionally excluded from this technique.

Although this work represents a proof-of-concept investigation based on a patient-specific simulation, the proposed methodology was intentionally developed using a simplified and reproducible model. While physical interactions such as attenuation and backscattering may vary across individual anatomical conditions, the dosimetric trends and design principles demonstrated in this study are broadly applicable. This adaptability supports the potential translation of the proposed multilayer applicator into routine contact IRT workflows, where patient-specific customization combined with standardized structural concepts may facilitate more personalized treatments while maintaining high dosimetric accuracy.

Overall, this study reinforces the importance of integrating 3D printing technologies and imaging-guided planning into modern IRT workflows. The precise control of catheter layout, shielding design, and patient-specific anatomical adaptation facilitates accurate dose delivery while minimizing collateral exposure. This personalized approach supports improved dosimetric outcomes and potentially better clinical and cosmetic results, as highlighted in the recent literature on IRT for complex superficial targets [17,19,20,21].

This work represents a preliminary in silico investigation aimed at evaluating the dosimetric impact of protective shielding integrated into multilayer applicators. The simplified phantom model ensured clarity and reproducibility while isolating the influence of shielding thickness and dose calculation algorithms. This approach allowed the identification of generalizable dosimetric patterns, independent of patient-specific variability, which can serve as a robust foundation for subsequent translational steps. Although it does not simulate the full anatomical variability encountered in clinical settings, such as tissue curvature, air-tissue interfaces, or irregular skin topography, these factors represent the logical next step toward clinical translation. Furthermore, the geometrical shielding model used in this study can be enhanced in future iterations through adaptive or flexible design, improving anatomical fit and patient comfort. In particular, the integration of biocompatible and radiotransparent materials could open new perspectives for clinical usability, facilitating both sterilization procedures and intraoperative handling. While catheter configuration was standardized and no dose optimization algorithms were applied, the use of patient-specific inverse planning remains a promising tool to further enhance dose conformity and safety in shielded IRT applications. Such an approach may enable tailored protection of organs at risk without compromising target coverage, which is especially relevant in cases of irregular lesions or when critical structures lie in close proximity to the treatment area. Future developments should include experimental validation through 3D-printing prototypes, mechanical testing to verify robustness and usability, and pre-clinical dosimetric measurements in anthropomorphic phantoms. These validation steps will also allow the assessment of workflow integration, including imaging compatibility, sterilization feasibility, and procedural ergonomics, thereby providing a comprehensive evaluation of clinical readiness. Subsequent steps will involve pilot patient applications under controlled clinical conditions to assess feasibility, tolerability, and reproducibility in real-world workflows.

Although this work is based on a simplified, homogeneous phantom model, the applicator geometry and catheter configuration were carefully designed to mimic the anatomical conditions and implantation techniques typically adopted in routine clinical contact IRT. Therefore, the dosimetric trends reported here provide meaningful insight into potential clinical benefits, particularly for challenging superficial lesions located near OARs. This is particularly relevant in clinical contexts such as dermatological tumors of the head and neck region, periorbital or auricular lesions, and pediatric indications where tissue sparing and cosmetic outcome play a pivotal role. In this perspective, a translational pathway moving from simulation to printing, phantom validation, and finally patient application will be essential to confirm the safety and effectiveness of shielded multilayer applicators before their integration into routine practice. Ultimately, such an approach may contribute to the expansion of IRT applications by offering more personalized, precise, and patient-centered treatments.

## 5. Conclusions

This study demonstrates the feasibility and dosimetric benefit of integrating high-density shielding into 3D-printed multilayer applicators for superficial IRT. The TG-186 algorithm provided a more accurate estimation of the dose to OARs, revealing a dose sparing effect that became more evident with increasing shielding thickness. Such results, although obtained in a simplified simulation model, are consistent with the expected physical behavior of high-density materials and confirm the potential role of advanced dose calculation algorithms in capturing these effects. A range between 6 and 9 mm appears optimal, balancing target coverage, TW, and OAR protection. Within this range, shielding seems to maintain adequate CTV coverage while avoiding excessive increases in hotspot regions, suggesting a possible compromise between therapeutic efficacy and normal tissue protection. These results support personalized, image-guided IRT approaches. Future developments should explore fabrication, clinical validation, adaptive device design, and integration into routine workflows. Additional studies will also be required to evaluate the mechanical stability of the applicators and their usability in clinical settings in personalized applications.

## Figures and Tables

**Table 1:** Dosimetric results obtained with TG-43 and TG-186 calculations using the same multilayer catheter configuration, without additional shielding. Reported parameters include CTV coverage (V95%, V100%, V150%), OAR dose (D_2cc_), and TW.

	V_95%_ CTV (%)	V_100%_ CTV (%)	V_150%_ CTV (%)	D2cc OAR (%)	TW (mm)
TG-43	95.2	88.2	1.5	79.4	7.2
TG-186	92.6	84.2	1.5	76.5	7.0

## Data Availability

The original contributions presented in this study are included in the article. Further inquiries can be directed to the corresponding author.
